# Directly observed treatment short-course (DOTS) for treatment of new tuberculosis cases in Somali Regional State, Eastern Ethiopia: ten years retrospective study

**DOI:** 10.1186/s13104-015-1325-3

**Published:** 2015-08-19

**Authors:** Desalegn Woldeyohannes, Solomon Sisay, Belete Mengistu, Hiwot Kassa

**Affiliations:** Aklilu Lemma Institute of Pathobiology, Addis Ababa University, P.O.Box 1176, Addis Ababa, Ethiopia; Department of Clinical, John Hopkins University-TSEHAI Project, P.O.Box 5606, Addis Ababa, Ethiopia; Directorate of Pastoralist Health Promotion and Disease Prevention, Federal Ministry of Health, P.O.Box 1234, Addis Ababa, Ethiopia; Department of Nursing, College of Medicine and Health Sciences, University of Gondar, P.O.Box 196, Gondar, Ethiopia

**Keywords:** Case detection rate, Directly observed treatment short-course, New tuberculosis cases, Somali Region, Treatment success rate

## Abstract

**Background:**

A third of the world population is infected with tuberculosis (TB) bacilli. TB accounts for 25 % of all avoidable deaths in developing countries. The objective of the study was to assess impact of directly observed treatment short-course (DOTS) strategy on new tuberculosis case finding and treatment outcomes in Somali Regional State, Ethiopia from 2003 up to 2012 and from 2004 up to 2013, respectively.

**Methods:**

A health facility based retrospective study was employed. Quarterly reports were collected using World Health Organization (WHO) reporting format for TB case finding and treatment outcome from all zones in the region to the Federal Ministry of Health.

**Results:**

A total of 31, 198 all types of new TB cases were registered and reported during the period from 2003 up to 2012, in the region. Out of these, smear positive pulmonary TB cases were 12,466 (40 %), and 10,537 (33.8 %) and 8195 (26.2 %) for smear negative pulmonary TB and extra-pulmonary TB cases, respectively. An average case detection rate (CDR) of 19.1 % (SD 3.6) and treatment success rate (TSR) of 85.5 % (SD 5.0) for smear positive pulmonary TB were reported for the specified years period. For the overall study period, trend chi-squire analysis for CDR was *X*^2^ = 2.1; P > 0.05 and *X*^2^ = 5.64; P < 0.05 for TSR.

**Conclusions:**

The recommended TSR set by WHO was achieved (85.5 %) and the CDR reported was far below (19.1 %) from the recommended target. Extensive efforts should be established to maintain the achieved TSR and to increase the low CDR for the smear positive pulmonary TB cases through implementing alternative case finding strategies.

## Background

According to the World Health Organization (WHO) Global tuberculosis (TB) report 2012, there were an estimated 8.7 million incident cases and 12 million prevalent cases of TB globally, in 2011, of which 1.1 million (13 %) were among people living with human immunodeficiency virus (HIV) [1]. Although the number of TB cases was stable or falling in 5 of 6 WHO regions, it is growing in Africa where the TB epidemic is still driven by the spread of HIV [2]. The HIV pandemic presents a massive challenge to global TB control with a co-infection prevalence rate of 5 % or more in 8 African countries [3].

According to WHO global TB report in 2012, which considered the findings from the national TB prevalence survey, there were an estimated 220,000 (258 cases per 100,000 populations) incident cases of TB in Ethiopia in 2011. The same report mentioned that the prevalence of TB was estimated to be 200,000 (237 cases per 100,000 populations). Additionally, there were an estimated 15,000 deaths (18 cases per 100,000 populations) due to TB, excluding HIV related deaths in the country during the same period [4].

Directly observed treatment short-courses (DOTS) are both practical and effective for diagnosis, treatment, and monitoring of TB. Each of the five components of DOTS—political and administrative commitment; case detection, primarily by microscopic examination of sputum of patients presenting to health facilities; standardized short course chemotherapy given under direct observation; adequate supply of good quality drugs; and systematic monitoring and accountability for every patient diagnosed—is integral to success [5].

On the other hand, some studies didn’t detect the advantage of DOTS over self-administered treatment [6, 7]. In other studies, defaulting and poor methods of diagnosis, treatment and monitoring of treatment [8] were observed.

In Ethiopia, DOTS, which was fully implemented in the country in 2000, its coverage was 70 and 95 % in 2003 and 2004, respectively. Similarly, 90 % of the country’s population had true access to DOTS because of low health coverage [9]. Case detection rate in Ethiopia was 36 % in 2004 whereas treatment success rate for new smear positive cases in the DOTS strategy was 80 % in 2000 and dropped to 70 % in 2003 [2] with no obvious explanation for deterioration.

Even though it is critical to evaluate the impact of the program in different parts of the country, few similar researches were conducted in some parts of the country in which the findings revealed that there were more gaps in organizational issues such as misuse and under use of TB registration books, challenges in follow-up of TB patients, low case detection of TB and increased rate of treatment defaulter [10]. A study which investigated a 10-year experience of TB control program in Southern region of Ethiopia has shown weaknesses in organizational issues such as under use of the TB registry, deficient follow-up procedures, and alarming rates of defaulting [10]. The same result of low case detection rate (CDR) and treatment success rate (TSR) were also reported by Kidanemariam and Alemayehu [11, 12]. Similarly Woldeyohannes et al. [13] and Sisay et al. [14] reported low level of CDR in Addis Ababa and Gambela regions, respectively. Therefore, this study aimed at evaluating the impact of the DOTS program on new TB case findings and their treatment outcome in Somali Regional State, Ethiopia.

## Methods

### Study design

Institutional based retrospective data was collected for TB cases who were registered and reported from 2003 up to 2012 and for their treatment outcome from 2004 to 2013 in Somali Regional State.

### Study area

According to the 2007 report from Central Statistical Agency (CSA) of Ethiopia, Somali Regional State had a total population of 4,445,219. Out of whom, males and females consisted of 2,472,490 (55.6 %) and 1,972,729 (44.4 %), respectively. Urban populations were estimated to be 623,004 (14.02 %), while 1,687,858 (37.97 %) were pastoralist populations. With an estimated area of 279,252 square kilometers, the regional state had an estimated density of 15.9 persons per square kilometer. For the entire regional state, 685,986 households were counted, which indicated that an average of 6.8 persons was residing in a single household. The average urban and rural household holding capacities were 6 and 6.5 persons, respectively. The regional state is divided into 9 administrative zones and 53 districts [15].

#### Source population

All new TB patients in the region.

#### Study population

New TB patients who were registered during the study period.

### Inclusion and exclusion criteria

All forms of TB cases registered during the study period were included in the study. Treatment outcome of smear-positive pulmonary tuberculosis cases were focused and evaluated as smear-positive TB cases are the main sources of infection for community. Treatment outcome of extra-pulmonary tuberculosis and smear-negative pulmonary tuberculosis cases were excluded as data for their treatment outcome are not explicitly completed.

### Data collection methods and procedures

WHO standardized reporting formats as instrument of data collection tools were used to collect the necessary data on case detection and treatment outcome. Data were first collected from health facilities (HF) where TB focal persons compiled the data from registration books, report and submit it on quarterly basis to zonal TB focal persons, and in turn, the zonal TB focal persons submit report to regional TB program officer who frequently checked for completeness, quality and accuracy of reports. Finally, summarized, analyzed, interpreted and compiled data report were sent to the National Tuberculosis and Leprosy Control Program (NTLCP) of Federal Ministry of Health (FMoH) by regional TB program officer. Trained data collectors and investigators collected data reported from all DOTS implementing health facilities in all zones of the region. Data collectors were participated in collecting information from TB registration books in the facilities by using WHO reporting formats through cross-checking the reports which were made by TB focal persons for the purpose of assuring quality of data. Data collectors also collected summary data of all zonal reports to Regional Health Bureau by comparing data which were previously reported by zonal TB focal persons. Similarly, data were collected from the Regional Health Bureau by looking through the reports which were made by the regional TB focal person to the FMoH.

### Data validation

Currently, FMoH is implementing Health Management Information System (HMIS) for all health program including TB for recording and reporting purposes, which consisted of its own unique data collection book (registration book) and reporting format. Data which were obtained by WHO reporting formats for TB case detection and treatment for the study period were crosschecked for the same information which was reported through HMIS for maintaining accuracy of the reporting data for the same study period.

### Operational definitions

#### Case definition

Case detection rate: Percentage of smear-positive TB cases detected among the total number of TB cases estimated to occur. Estimation was made by WHO estimating factor for each reporting year for the study period by considering the incidence rate of TB from the total of 100,000 populations.

New case: A patient who has never had treatment for TB or has been on anti-TB treatment for less than 4 weeks.

Relapse: A patient who has been declared cured or treatment completed from any form of TB in the past but found to be smear-positive or culture positive.

Return after default: A patient who had previously been recorded as defaulted from treatment and returns to the health service with smear-positive sputum.

#### Treatment outcome

Treatment success rate: the sum of cure rate and completion rate.

Defaulter: A patient who has been on treatment for at least 4 weeks and whose treatment was interrupted for 8 or more consecutive weeks.

Transfer out: A patient who has started treatment and has been transferred to another health facility and for whom treatment outcome is not known at the time of evaluation.

Cured: A patient who is sputum smear-negative one month prior to the completion of treatment and on at least one previous occasion (usually at the end of the 2nd or 5th month).

Treatment completed: A patient who has completed treatment but in whom smear result are not available at or one month prior to the completion of treatment.

Treatment failure: A patient who remained smear positive or became again smear positive at the end of five months or later after commencing treatment.

Died: A patient who dies for any reason during the course of treatment.

Treatment success rate: A sum of TB cases who completed treatment and who declared cured.

### Data analysis

SPSS version 20.0 packages were used to analyze and interpret the data. We entered the variables in SPSS based on reporting years, case detection rate, treatment outcome (cured, death, failure, treatment success rate, relapse, and default etc.). Data were summarized using frequencies, percentages and standard deviations including mean values. Trends of TB cases, CDR and TSR were analyzed using chi-squire for trends.

### Ethical considerations

The study secured ethical approval from Somali Regional State Health Bureau Ethical Clearance Committee. Consent was requested from Somali Regional State Health Bureau and FMoH in order to get the necessary secondary data of all DOTS implementing facilities in the zones of the region.

## Results

A total of 31,198 all types of new TB cases were registered and reported from 2003 to 2012 in the study area. Out of these, smear-positive pulmonary TB cases were 12,466 (40 %), and 10,537 (33.8 %) and 8195 (26.2 %) for smear-negative pulmonary TB and extra- pulmonary TB cases, respectively. The highest all new TB cases were registered in the year 2010 and lowest in the year 2009 with a total number of 4543(14.5 %) and 1977(6.3 %), respectively (Table 1). There was a consistent increase trend in the number of all new TB cases from 2602 in the year 2005 to 4543 in 2010 (Trend *X*^2^ = 4.5; P < 0.05).

**Table 1:** All new TB case findings in Somalia Region from 2003 to 2012

Year	Smear positive PTB (N)	%	Smear negative PTB (N)	%	Extra Pulmonary TB (N)	%	All forms of TB (N)
2012	902	31.3	1141	39.7	835	29.0	2878
2011	769	39.0	753	38.0	455	23.0	1977
2010	1439	31.7	1850	40.7	1254	27.6	4543
2009	1495	34.8	1716	40.0	1083	25.2	4294
2008	1406	42.8	1037	31.6	838	25.6	3281
2007	1249	40.0	1121	35.7	761	24.3	3131
2006	1381	44.6	882	28.5	834	26.9	3097
2005	1166	44.8	677	26.0	759	29.2	2602
2004	1289	46.1	759	27.1	749	26.8	2797
2003	1370	52.8	601	23.1	627	24.1	2598
Total	12,466	40.0	10,537	33.8	8195	26.2	31,198

From the year 2006 up to 2012, a total number of 7737 new smear positive pulmonary TB cases were registered. And out of these, 4678 (60 %) were males and the rest were females. The highest numbers [2436 (31.4 %)] of new smear positive TB cases were registered in the age group of 15–24 and followed by 2002 (26 %) in the age group of 25–34, while the least new smear positive TB cases [41 (1 %)] were registered in the age group of 0–14 (Table 2).

**Table 2:** Total number of smear positive new TB cases by sex and age group in Somalia Regional State from 2006 to 2012

Age	Sex				Total	
	Male		Female		No.	%
	No.	%	No.	%		
0–4	22	54	19	46	41	1
5–14	169	49	173	51	342	4
15–24	1481	61	955	49	2436	31.4
25–34	1213	61	789	49	2002	26
35–44	736	59	518	41	1254	16
45–54	507	61	321	59	828	11
55–54	312	62	190	48	502	7
65+	238	74	94	36	332	4
Total	4678	60	3059	40	7737	100

**Fig. 1:**
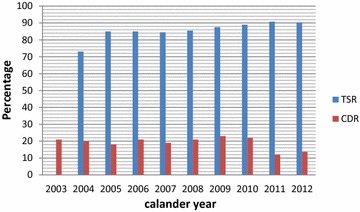
Trend of CDR and TSR in Somalia Region from 2003 to 2012

The average CDR for new smear positive pulmonary TB cases was found to be 19.1 % for the overall study period, on the other hand, the lowest CDR (12 %) and the highest CDR (23 %) were observed in the years of 2011 and 2009; respectively (Fig. 1), with the overall study period Trend *X*^2^ = 2.1; P > 0.05.

A total number of 12,466 smear-positive pulmonary TB cases were registered for the study period. Of them, 12,041(93.6 %) were evaluated for their treatment outcomes from 2004 up to 2013 in the region. Among the total evaluated smear positive TB cases, an average cure rate of and TSR were found to be 81.9 and 85.5 %, respectively. TSR (85.5 %) were calculated by adding cure rate (81.9 %) and treatment complete rate (3.6 %) for the overall study period. The lowest (73.1 %) and the highest (90.8 %) TSRs were observed in 2004 and 2011; respectively, with the overall study period Trend *X*^2^ = 5.64; P < 0.05.

As far as death rate, treatment failure rate, defaulter and transfer out rates are concerned, they were found to be 4.5, 0.8, 4.2 and 4.0 % for the study period, respectively (Table 3). In the study period, the cure rate and treatment success rate increased from 82.9 to 86.2 % and from 84.4 to 90.2 %, respectively. The death rate and defaulter rate had increases from 2.9 to 5.4 % with average value of being 4.5 % (SD 1.1) and from 2.3 % up to 4.7 % with average value of being 4.2 % (SD 1.03) over the study period, respectively. Moreover, treatment failure rate and transfer out rate fluctuated over the study period with the average value of being 4.2 and 4.0 %, respectively (Table 3).

**Table 3:** Treatment outcome of smear positive pulmonary TB cases in Somalia region from 2004 to 2013

Year	Registered (N)	Evaluated (N)	Cure (%)	Treatment completed (%)	Treatment success (%)	Death (%)	Failure (%)	Default (%)	Transfer out (%)
2013	769	769	86.2	4.0	90.2	2.9	0.6	2.3	4
2012	1439	1439	87.0	3.8	90.8	3.0	0.5	2.7	3.0
2011	1495	1304	85.0	4.0	89.0	3.1	0.5	3.5	4.1
2010	1406	1364	80.8	6.7	87.5	5.0	0.7	4.5	2.1
2009	1249	859	81.8	3.6	85.4	5.8	0.0	4.9	3.8
2008	1381	1307	78.2	6.3	84.5	4.7	1.2	4.7	4.7
2007	1166	1166	82.6	2.4	85.0	4.8	1.0	4.8	4.4
2006	1289	1166	82.6	2.4	85.0	4.8	1.3	4.5	4.4
2005	1370	1370	72.1	1.0	73.1	5.5	0.8	5.5	5.2
2004	1297	1297	82.9	1.5	84.4	5.4	1.1	4.7	4.3
Total	12,861	12,041	81.9	3.6	85.5	4.5	0.8	4.2	4.0

## Discussion

The CDR for new smear positive pulmonary TB cases for the overall study period was found to be 19.1 %. The overall low CDR relative to the WHO target of 70 % might be as the result of CDR relies and calculated based on the estimated prevalence in the country as denominator and that estimated prevalence is high making the CDR low. It is, therefore, difficult to measure accurately in most settings especially in the context of a high prevalence of HIV [16]. Other possible reasons might be due to inadequate decentralization of DOTS program, shortage of resource and trained personnel and low sensitivity of smear microscopy. Likewise, researches carried out in different regions of Ethiopia reported different case detection rates. Low CDRs were reported in Oromia by Kidanemariam et al. (31.7 %) [11], Alemayehu et al. (23.7 %) in Benshangul [12] and Sisay et al. (40.9 %) in Gambela region [14]. And, relatively higher CDR (68 %) was reported by Woldeyohannes et al. in Addis Ababa, Ethiopia [13]. For Africa the CDR was reported to be 63 % while that of the world for all forms of TB in 2012 was reported to be 66 % [1]. The difference in reported CDR could be attributed to the calculation for CDR done based on WHO estimate, burden of HIV, different population size between studies, and TB diagnostic capabilities of the region etc.

An average value of TSR of 85.5 % was achieved for the overall study period, which was almost similar to that of WHO target and national average 84 % [17] and was higher than Africa 72 % [2]. In studies from different parts of Ethiopia, a relatively lower TSRs of 80 % [13], 55.7 % [14], 81 % [11], 66.41 % [12], 80 % [18] and 80.5 % [19] were reported. The high TSR reported in the study might therefore suggest good performance by the Region’s TB program in the areas of adherence and follow up of patients to the course of treatment, relatively proper recording and reporting system, adequate treatment regimens and decrease in incidence of drug resistant strains, and government commitment to ensure comprehensive TB control activities [20]. In other similar studies which were carried out in Enugu and Ebonyi States in Nigeria reported TSR of 82 and 80 %, respectively [21, 22]. However, a relatively higher TSR (88.2 %) was reported from a five year retrospective study done in Mersin, Turkey [23]. In the current study, TSR for the years (2004–2013) varied from 73.1 to 90.8 %. The reasons for the disparities should be explored by the Region TB program so as to improve the disease control.

The cure rate accepted as safer and more valuable indicator than treatment success rate [24] was approximately 80 % in the world at large, 60 % in Europe and Turkey between 2005 and 2008, and has been noted to increase since [25] and 57.88 % in Mersin, Turkey [23] and (68.6 %) in one retrospective study (1997–2011) carried out in Arsi Zone, Central Ethiopia [19]. In our study, it was observed that the cure rate is relatively higher than in Europe, Mursin/Turkey and Arsi Zone, Central Ethiopia and almost equal to in the world at large. In this study during the study period, the reduction in the death rate, treatment failure rate, default rate, and transfer out rate had significant effects on the increase of treatment success and cure rate.

Treatment completion rate was reported to be 7 % in the world between 2005 and 2008 and remained constant, reduced to 8 from 13 % in Europe and to 33 from 44 % in Turkey, respectively [25] and 30.38 % in one retrospective study (2004–2008) carried out in Mersin, Turkey [23] and 16.2 % in study carried out in Arsi Zone, Central Ethiopia. In our study, treatment completion rate in Somalia was relatively lower than the value in the world, Europe, Turkey and report from Arsi Zone, Central Ethiopia. Again, in our study, while treatment completion rate increased during the 10-year period which is important in terms of indicating a reduction in the number of microbiological analyses rate at the beginning and end of therapy.

A 0.8 % of average treatment failure rate among smear positive pulmonary TB cases was reported. In other three similar studies done in different regions of Ethiopia, the same low treatment failure rates were reported [11, 13, 19]. In study carried out in Mersin, Turkey [23] reported a relatively higher average treatment failure rate of 4.26 %. The lower treatment failure rate in our study could be due to the overall low prevalence of multi drug resistant tuberculosis (MDR TB) in Ethiopia which was 1.6 % at national level [26] and 1.1 % in Eastern Ethiopia [27] for new smear positive cases.

Secondary data from the Region’s TB program was used for the study; therefore, minimal errors could have occurred during data entry and computations but would not have affected the study’s results. In addition, DOTs coverage for each year of the study period was not available which prevents the study from addressing the significant of expansions of the program on the current CDR and TSR. Also, the periods of years reviewed for all study objectives were not uniform (from 2003 to 2006, for example, for new smear-positive pulmonary TB cases) because of incomplete data, which limited trend assessments for such variable.

## Conclusions

The recommended treatment success rate set by WHO was achieved (85.5 %) and the reported CDR was very low (19.1 %). Efforts should be there to maintain the achieved TSR and to increase low CDR for smear-positive pulmonary TB cases observed in the region.

## Abbreviations

CDR: case detection rate
CSA: Central Statistical Agency
DOTS: directly observation treatment short-course
FMoH: Federal Ministry of Health
HF: health facility
HIV: human immunodeficiency virus
HMIS: Health Management Information System
MDR-TB: multi drug resistant tuberculosis
NTLCP: National Tuberculosis and Leprosy Control Program
PTB: pulmonary tuberculosis
SD: standard deviation
SPSS: Statistical Package for Social Science
TB: tuberculosis
TO: transfer out
TSR: treatment success rate
WHO: World Health Organization
